# Single cell assessment of yeast metabolic engineering for enhanced lipid production using Raman and AFM-IR imaging

**DOI:** 10.1186/s13068-018-1108-x

**Published:** 2018-04-10

**Authors:** Kamila Kochan, Huadong Peng, Bayden R. Wood, Victoria S. Haritos

**Affiliations:** 1 Centre for Biospectroscopy, School of Chemistry, Monash University, Clayton Campus, Clayton, VIC 3800 Australia; 2 Department of Chemical Engineering, Monash University, Clayton Campus, Clayton, VIC 3800 Australia

**Keywords:** Lipid bodies, Spectroscopy, Metabolic engineering, Heterogeneity, Triacylglycerol, Fatty acids

## Abstract

**Background:**

Biodiesel is a valuable renewable fuel made from derivatized fatty acids produced in plants, animals, and oleaginous microbes. Of the latter, yeasts are of special interest due to their wide use in biotechnology, ability to synthesize fatty acids and store large amounts of triacylglycerols while utilizing non-food carbon sources. While yeast efficiently produce lipids, genetic modification and indeed, lipid pathway metabolic engineering, is usually required for cost-effective production. Traditionally, gas chromatography (GC) is used to measure fatty acid production and to track the success of a metabolic engineering strategy in a microbial culture; here we have employed vibrational spectroscopy approaches at population and single cell level of engineered yeast while simultaneously investigating metabolite levels in subcellular structures.

**Results:**

Firstly, a strong correlation (*r*^2^ > 0.99) was established between Fourier transform infrared (FTIR) lipid in intact cells and GC analysis of fatty acid methyl esters in the differently engineered strains. Confocal Raman spectroscopy of individual cells carrying genetic modifications to enhance fatty acid synthesis and lipid accumulation revealed changes to the lipid body (LB), the storage organelle for lipids in yeast, with their number increasing markedly (up to tenfold higher); LB size was almost double in the strain that also expressed a LB stabilizing gene but considerable variation was also noted between cells. Raman spectroscopy revealed a clear trend toward reduced unsaturated fatty acid content in lipids of cells carrying more complex metabolic engineering. Atomic force microscopy-infrared spectroscopy (AFM-IR) analysis of individual cells indicated large differences in subcellular constituents between strains: cells of the most highly engineered strain had elevated lipid and much reduced carbohydrate in their cytoplasm compared with unmodified cells.

**Conclusions:**

Vibrational spectroscopy analysis allowed the simultaneous measurement of strain variability in metabolite production and impact on cellular structures as a result of different gene introductions or knockouts, within a lipid metabolic engineering strategy and these inform the next steps in comprehensive lipid engineering. Additionally, single cell spectroscopic analysis measures heterogeneity in metabolite production across microbial cultures under genetic modification, an emerging issue for efficient biotechnological production.

**Electronic supplementary material:**

The online version of this article (10.1186/s13068-018-1108-x) contains supplementary material, which is available to authorized users.

## Background

Biodiesel is a versatile renewable fuel composed of fatty acid methyl esters produced from animal and plant lipids-triacylglycerols (TAGs) [1, 2]. Microbial oils also have advantages as a source of TAG: microorganisms can be grown on non-food sugars, are minimally affected by seasons, generally robust and have short life cycles [1]. Many types of oleaginous eukaryotic species accumulate TAG in lipid bodies (LB), intracellular organelles considered primarily as storage vesicles for neutral lipids, in amounts up to 70% of total DCW biomass [1, 3, 4]. However, for cost-effective and high yielding production of TAG in oleaginous organisms such as yeast, metabolic pathway engineering and maximizing yield across the cultures is necessary [1, 3, 5, 6].

The metabolic engineering strategies for improvement of lipid production include up and down regulation of genes involved in the main steps of yeast lipid production: fatty acid (FA) biosynthesis, lipid accumulation and sequestration [1, 7]. Increased fatty acid production can be achieved by higher expression of aldehyde dehydrogenase (ALD), acetyl-CoA synthetase (ACS) [8] and acetyl-CoA carboxylase (ACC) [9, 10] and FA accumulation in TAG is catalyzed by diacylglycerol acyltransferase (DGAT) [7] and other acyltransferases. Heterologous expression of highly active acyltransferases such as the DGAT1 from *Arabidopsis thaliana* has been shown to increase lipid yield in the yeast *Saccharomyces cerevisiae* [11] and similarly, LB stabilization proteins such as caleosin (AtClo1) [12] have been shown to support LB formation. Down regulation of lipid mobilization enzymes such as the major TAG lipase, Tgl3, in *S. cerevisiae*, can also increase lipid content of cells [13].

Another factor affecting the productivity of microbially produced lipids is heterogeneity in individual cell performance across a culture [14]. Some of the variance is due to the stochastic nature of biological processes within gene regulation, transcription and translation, but response can be further distorted by variable responses to environmental conditions [15] including genetic engineering. While heterogeneity in cellular processes among isogenic microbial cells in a population has been known for some time, recent developments in visualization technologies have allowed measurement and monitoring of metabolite production both of populations and single cells within.

The formation and growth of LBs in cells are most commonly analysed by fluorescence imaging or histochemical staining [16]; these enable the visualization of LBs in multiple cells at once, but without providing details of their chemical composition. Vibrational spectroscopy-based techniques such as infrared (IR) and Raman spectroscopy (RS) are used in combination with digital imaging to enable rare insights into single cells of microalgae and yeast [17–20]. In particular, confocal Raman spectroscopy (CRS) has revealed intracellular structures such as LBs, due to its high spatial resolution falling in the range of few hundred nm depending on the excitation wavelength [21] and chemical composition information of selected intracellular structures [18, 19, 22]. The spatial resolution of imaging with the use of conventional IR spectroscopy is restricted by wavelength diffraction spatial resolution limit (~ 5.5 µm), making it less suitable for the study of intracellular structures of microorganisms. Recently, however, a novel technique has been developed, based on a combination of IR spectroscopy and Atomic Force Microscopy (AFM–IR). In AFM–IR, IR absorption spectrum is acquired indirectly, by measuring the resulting thermal expansion of the sample. This approach overcomes the limitation of conventional IR-based imaging and achieves a spatial resolution of ~ 100 nm, thus enabling analysis of cellular constituents at the single cell level.

The aims of this research were to determine the effectiveness of metabolic pathway engineering approaches for enhanced lipid content in yeast through an examination of subcellular structures including LBs and to examine the heterogeneity of response in *S. cerevisiae* yeast cultures via vibrational spectroscopic approaches. We measured the overall changes in lipid content and other metabolites at a population level in whole cells by ATR-FTIR following the introduction of genetic modifications, then undertook detailed studies of subcellular structures and changes in their chemical composition in single cells using CRS and AFM–IR. These approaches provided detailed, single cell information on subcellular structures at high-resolution for the engineered strains and also allowed representative sampling of the population for these traits. The increase in the total lipid content of engineered cells demonstrated in bulk samples reflected either a greatly increased number or greatly increased size of LBs; the increased size was likely due to the influence of the LB stabilizing protein, caleosin, expressed in these cells. In engineered yeast, LB fatty acid composition shifted towards lower content of UFA relative to SFA in the highly lipidic strains and cytoplasmic carbohydrate stores were heavily reduced. Vibrational spectroscopy analysis of yeast cells revealed unprecedented information on the effectiveness and effects of metabolic engineering strategies for higher lipid content that will also guide future approaches to the field.

## Methods

### Yeast cell lines and culture conditions

Four engineered strains of *S. cerevisiae* were evaluated in the study and compared with a control strain (CON) BY4741 (*MATa his3Δ1 leu2Δ0 met15Δ0 ura3Δ0)* transformed with three empty vectors. Genes selected for metabolic engineering are given in Table 1; the full description of the metabolic engineering strategy and selection and the combination of genes and expression plasmids are given in Ref. [23]. Expression of the introduced genes *AtDGAT1, SEACS*^*L641P*^*, ACC1*^*S659A, S1157A*^*, AtClo1* was regulated, respectively, by promoters GAL1, TEF1, PGK1 and GAL10.

**Table 1:** *Saccharomyces cerevisiae* strain names and introduced genes

Strain	Genes expressed
CON	Empty vectors^a^
HBY03	*AtDGAT1*
HBY14	*AtDGAT1 Tgl3Δ*
HBY20	*AtDGAT1 Tgl3Δ Ald6 SEACS* ^*L641P*^
HBY31	*AtDGAT1 Tgl3Δ Ald6 SEACS* ^*L641P*^ *ACC1* ^*S659A, S1157A*^ *AtClo1*

Δ indicates endogenous gene was knocked out

^a^pSP-GM2, pIYC04, pESC-leu2d

*Saccharomyces cerevisiae* were maintained based on their auxotrophy using yeast synthetic complete (SC) minimal medium, containing 6.7 g/L of yeast nitrogen base, 20 g/L glucose as well as a mixture containing appropriate nucleotide bases and amino acids for the various dropout options (SC-Leu, SC-His-Ura, SC-His-Leu-Ura). All strains were stored in 15% glycerol at − 80 °C before being cultured in 5 mL yeast SC minimal medium and incubated at 30 °C, 250 rpm, overnight. The culture OD600 nm was diluted to approximately 0.4 into 50 mL of SC induction medium containing 2% (w/v) galactose and 1% (w/v) raffinose in 250 mL flasks. The flasks were capped with aluminum foil and incubated at 30 °C, 250 rpm until cells were harvested at 72 h for the following analysis.

### GC measurements of total fatty acid content

Harvested cells were pelleted by centrifugation at 3000 rpm for 5 min, frozen at − 80 °C for ~ 1 h and subsequently freeze-dried overnight using a FreeZone^®^ 4.5 Liter Freeze Dry Systems (Labconco Corporation, USA) to obtain the dry cell weight (DCW) of each culture. Dry cells (~ 20 mg) were treated with 2 mL methanol/hydrochloric acid/chloroform (10:1:1, v/v/v) and heated at 90 °C for 1 h in sealed test tubes to convert lipids to fatty acid methyl ester (FAME). FAME was washed with 0.9% NaCl solution (1 mL) and extracted with hexane after mixing. FAME samples (1 µL) were analyzed by Agilent 7890A gas chromatography with flame ionization detection (GC-FID) as described previously [11].

### Lipid body visualization using confocal fluorescence microscope

Imaging of lipid droplets after Nile red (Sigma-Aldrich, USA) staining of unfixed stationary phase yeast cells was undertaken 72 h after induction of gene expression [11, 24]. 1 mL of harvested cells were transferred into a 1.5 mL reaction tube and washed twice with 1 mL sterile 50 mM Tris–HCl (pH 7.5). 1 µL of Nile Red stock solution was added into the cell suspension to obtain the final concentration of 1 μg/mL, gently mixed, incubated for 20 min at room temperature and centrifuged at 1000*g* for 2 min. 1 μL of dense cell suspension was mounted on a standard microscope slide and imaged by a Leica Microsystems SP5 confocal microscope coupled with HCX PL APO 63×/1.4 OIL CS oil-immersion objective in Monash Micro Imaging, Monash University and the data collected by Leica LAS X (Leica Microsystems, Inc.) microscope control software.

### Sample preparation for vibrational spectroscopy-based techniques

Yeast cells in phosphate buffered saline (PBS) were collected by centrifugation (1000 rpm, 5 min) and the pellet resuspended in 500 µL of ultrapure water, gently mixed and again centrifuged. This step was repeated 3 times to ensure removal of any residual PBS. The final pellet was resuspended in 500 µL of ultrapure water. For each yeast strain, 100 µL of the suspension was placed on each of two CaF_2_ slides and air-dried to obtain a dispersed layer of single cells. From each set of two samples, one was subjected to Raman measurements and the other was mounted on a flat magnetic stainless-steel substrate and designated for AFM–IR measurements. The remaining suspension (300 µL) was centrifuged and the pellet was placed directly on the Attenuated Total Reflection (ATR) crystal.

### ATR-FTIR measurements

ATR-FTIR data were recorded using a Bruker Alpha FTIR (Ettlingen, Germany) spectrometer with an ATR sampling device containing a single bounce diamond internal reflection element and equipped with a globar source, KBr beam splitter and a deuterated triglycine sulfate detector. Spectra were recorded at a resolution of 6 cm^−1^ in the spectral range of 4000–900 cm^−1^. For each strain, 3 biological replicates were studied and for each of these, 3 technical replicates were recorded (*n*_single_strain_ = 9, *n*_total_ = 45). Background spectra were collected directly prior to each measurement (64 scans). After recording the background, 0.5 µL of yeast pellet was placed on the crystal and air-dried for approximately 10 min. Each spectrum was recorded using 64 co-added interferograms.

### Raman measurements

Raman measurements were collected using WITec confocal CRM alpha 300 Raman microscope (WITec, Ulm, Germany). The spectrometer was equipped with an air-cooled solid-state laser operating at 532 nm, a CCD detector, cooled to − 60 °C and 600 grooves/mm grating. The laser was coupled to the microscope via an optical fiber with a diameter of 50 µm. For data collection, a dry Olympus MPLAN (100×/0.90NA) objective was used. The monochromator of the spectrometer was calibrated using Raman scattering line produced by a silicon plate (520.5 cm^−1^). For each strain, 3 biological replicates were studied and for each of these replicates, 6 individual cells were mapped (*n*_single_strain_ = 18, *n*_total_ = 90). The size of mapped area was adapted individually, depending on the size of the cell. Data were collected in the spectral range of 3705–0 cm^−1^, with the spectral resolution of 3 cm^−1^. The integration time for a single spectrum was 0.1–0.3 s. Laser power was adjusted individually for each sample, not exceeding the range of 5–7 mW. Raman measurements and initial data analysis were performed using WITec software (WITec Plus, Ulm Germany). Raman images were constructed by integration of selected marker bands without any preprocessing. Cluster analysis was carried out after cosmic spike removal (CRR) and background subtraction (polynomial fit, 2nd order). The Raman data were analyzed with *k*-means Clustering (KMC) using the Manhattan distance and Ward’s algorithm.

### AFM–IR measurements

AFM–IR measurements were performed with a NanoIR2 system (Anasys Instruments Inc., Santa Barbara, USA). The IR source was an optical parametric oscillator (OPO) laser, producing a 10 ns pulse at a 1 kHz repetition rate. A silicon cantilever (AppNano, Mountain View, CA 94043, USA) was used with nominal radius of 10 nm and a nominal spring constant of 0.5 N/m. The system was purged with N_2_ to control humidity. For each strain, 3 biological replicates were studied, with 6 single cells investigated for each biological replicate (*n*_single_strain_ = 18, *n*_total_ = 90). All single spectra were collected in the range of 1800–900 cm^−1^ with a spectral resolution of 8 cm^−1^ and IR maps at fixed wavenumber values, to investigate the distribution of selected components (specific wavenumber values are given in Results). Simultaneously to each IR map, the AFM height and deflection images were acquired. The maps were subsequently combined in MatLab (Mathworks, Natick, USA), PLS_toolbox (Eigenvector research, Manson, USA) and analyzed using *k*-means clustering to identify the presence and location of LBs. Following this analysis, single spectra were recorded from cytoplasm and LBs. All presented single spectra were normalized using the Standard Normal Variate (SNV) method and smoothed using the Savitzky-Golay algorithm with 13 smoothing points.

### Statistical tests

Absorbance data obtained for the engineered and control yeast strains were assessed for statistical significance by one way analysis of variance (ANOVA) at *p* < 0.01, *α* = 0.05; where significant differences were indicated, Student’s *t* test was applied the post hoc to data for engineered strains compared to control.

## Results and discussion

### Total fatty acid content: correlation between GC-FAME and ATR-FTIR spectroscopy

**Fig. 1:**
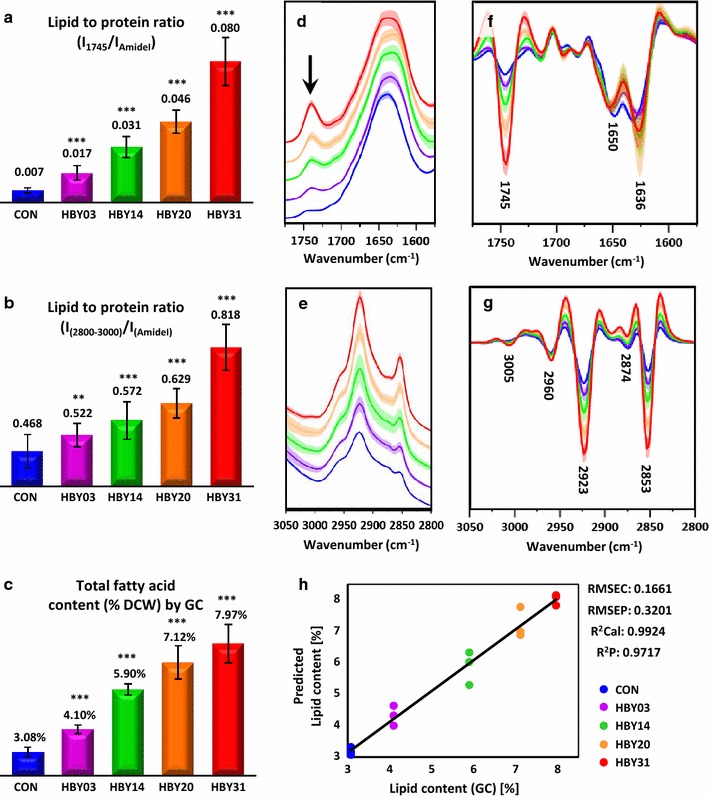
Results of the assessment of total fatty acid content obtained via (**a**, **b**, **d**–**h**) ATR-FTIR and (**c**) GC for all studies cell lines (CON, HBY03, HBY14, HBY20 and HBY31). (**a**, **b**) The ratio of lipid to protein obtained on the basis of ATR-FTIR spectra calculated as the ratio of: **a** the band at 1745 cm^−1^ to the amide I band and **b** the bands in high wavenumber region to the amide I band. **c** The total fatty acid content obtained for all cell lines through GC. Detailed fatty acid quantification from GC is presented in Additional file 1: Table S1. All bar charts (**a**–**c**) show the results obtained together with their standard deviation (SD). **d**, **e** Average ATR-FTIR spectra (with SD) of all cell lines and **g**, **h** their 2nd derivatives (with SD) used for calculation of ratios presented in (**a**) and (**b**) are shown in the range (**d**, **f**) 1550–1800 cm^−1^ and (**e**, **g**) 2800–3050 cm^−1^. Average ATR-FTIR spectra in the whole measured range (3600–600 cm^−1^) are presented in Additional file 1: Fig. S1, S2. **h** Results of prediction of the total fatty acid content for the validation set by the PLS regression model (2800–3050 cm^−1^) on the basis of 2nd derivatives of ATR-FTIR spectra. ***p < 0.01 vs control and **p < 0.05 vs control

The cell lines were firstly characterized using gas chromatography (GC) and ATR-FTIR spectroscopy, to provide an overview of the total fatty acid content (Fig. 1). As can be seen from the GC results (Fig. 1c), the total fatty acid content increased in all modified cell lines compared to control. The most significant increase was observed in the HBY31 cell line carrying alterations in 6 lipid-modifying enzymes, and the increases in lipid were proportional to the number of introduced genes. The same trend was observed in the ATR-FTIR spectra, particularly using the spectral ranges between 1800 and 1500 (Fig. 1d, f) and 3050–2800 cm^−1^ (Fig. 1e, g).

The first region (1800–1500 cm^−1^) includes the band at 1745 cm^−1^ (Fig. 1d, black arrow), attributed to stretching of C=O groups of lipids, whereas the second one (2800–3050 cm^−1^) contains a variety of bands originating from stretching of CH_2_ and CH_3_ groups in lipid chains [25]. Increase in the intensity of the band at 1745 cm^−1^ as well as the bands in the high wavenumber region between different cell lines is clearly visible (Fig. 1d–g) and follows the same trend as an increase in the total fatty acid content assessed from GC (Fig. 1c). Calculation of the ratio of those bands to the amide I band (Fig. 1a, b) enabled the visualization of changes in the lipid to protein ratio [26] in the studied cell lines, as it represents the total fatty acid content in the dried mass of cells. A PLS regression model, built on the basis of 2nd derivatives of ATR-FTIR spectra in the high wavenumber region, enabled the prediction of total fatty acid content (as a percentage of dry cell weight). The correlation between predicted and assessed from GC total fatty acid content for the validation set, together with the parameters of the PLS regression model, is shown in Fig. 1h. This approach provides a fast and straightforward measure of the effectiveness of lipid metabolic engineering strategy. The 2nd derivatives of ATR-FTIR spectra revealed significant variation in the band at 1636 cm−1 (Fig. 1f) assigned to deformation vibration of water, most likely due to intracellular water [27]. No correlation was observed between this and any of the lipid-related bands.

Our initial analysis of metabolically engineered cells undertaken on populations of whole cells addressed the basic question of lipid production between strains and demonstrated the power and rapid analysis of vibrational spectroscopy similar to that previously reported for naturally occurring yeasts by Ami et al. [28]. However, to enable us to address questions relating to subcellular structures and their chemical components and in regard to cell-to-cell variability within metabolic engineered cells required spectroscopic approaches with greater spatial resolution power.

### High-resolution CRS visualization and chemical analysis reveals subcellular structures and the impact of metabolic engineering

**Fig. 2:**
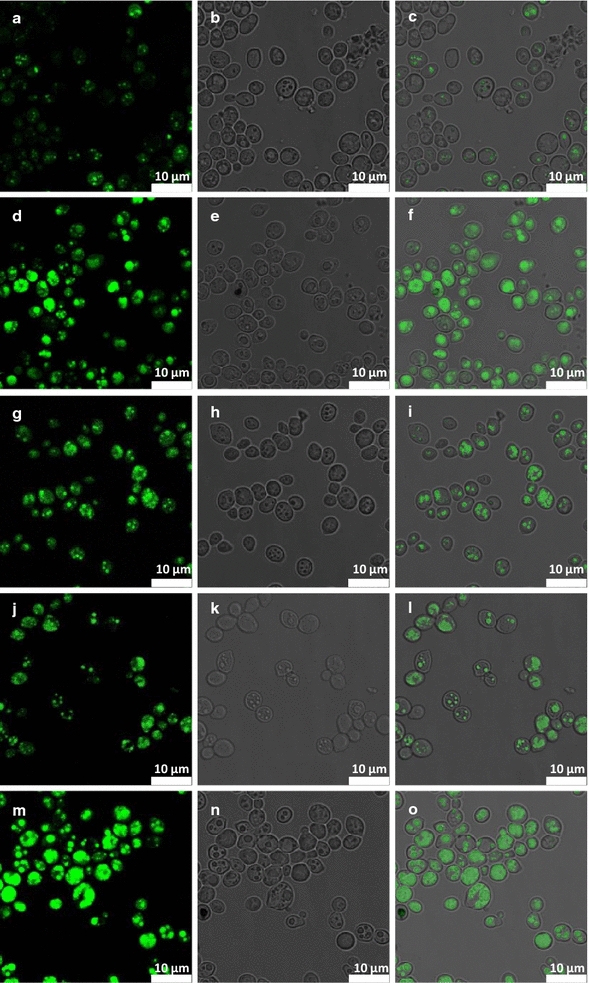
Images showing: (**a**, **d**, **g**, **j**, **m**) fluorescence results, (**b**, **e**, **h**, **k**, **n**) visible image in transmission and (**c**, **f**, **i**, **l**, **o**) overlay of visible and fluorescence image obtained for the: (**a**–**c**) CON, (**d**–**f**) HBY03, (**g**–**i**) HBY14, (**j**–**l**) HBY20 and (**m**–**o**) HBY31 yeast cell line at 72 h culturing. The scale bar is presented at the bottom right corner of each image

**Fig. 3:**
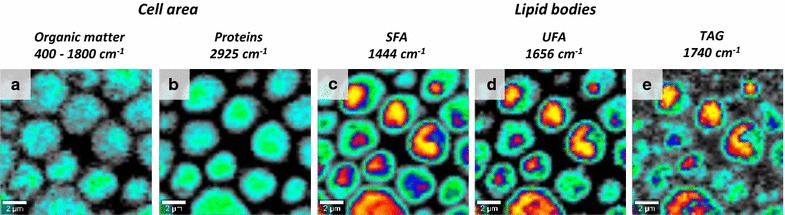
Distribution of selected components in HBY31 cells obtained by confocal imaging via Raman spectroscopy. The cell area was visualized through the integration of: **a** all bands in the fingerprint region (‘*Organic matter*’, 1800–400 cm^−1^) and **b** integration of the band corresponding to ν_s_(CH_2_) of proteins (‘*Proteins’*, 2925 cm^−1^). LBs can be visualised by integration of lipid-related bands corresponding to: **c** δ(CH_2_) in saturated fatty acids (‘*SFA*’, 1444 cm^−1^), **d** ν(C=C) in unsaturated fatty acids (‘*UFA*’, 1656 cm^−1^) and **e** ν(C=O) in triglycerides (‘*TAG*’, 1740 cm^−1^). The obtained results enable not only to visualise LBs, but also to demonstrate the presence of SFA, UFA and TAG specifically within those structures, as well as show homogenous distribution of proteins in the cytoplasm. Size of the imaged area: 12.86 × 12.36 µm

To confirm the presence of lipid bodies and estimate their amount, fluorescence imaging of the Nile Red stained cell lines was performed (Fig. 2). The results confirm the presence of LBs in all studied cell lines, with a clear increase in number in the engineered lines compared to control. In addition, amongst the highest engineered line (HBY31), numerous LBs of significantly larger diameter were observed (Fig. 2) but without providing any information about the composition of LBs or other intracellular structures. By comparison high spatial CRS imaging of HBY31 cells visualized LBs via lipid-related bands at 1444 cm^−1^ (bending mode of CH_2_), 1656 cm^−1^ (stretching mode of C=C) or 1740 cm^−1^ (stretching mode C=O) and chemical content through spectra to reveal the presence of saturated (SFA, 1444 cm^−1^), unsaturated fatty acids (UFA, 1656 cm^−1^) as well as triglycerides (TAG, 1740 cm^−1^) (Fig. 3). These confocal spectra ensured the collection of data only from a selected plane of given thickness and thus provide information exclusively about LBs, without interference of lipids from the cytoplasm and cell wall. Cell-to-cell variability in the presence of key metabolites within HBY31 cells is evident within the sample as shown in Fig. 3.

**Fig. 4:**
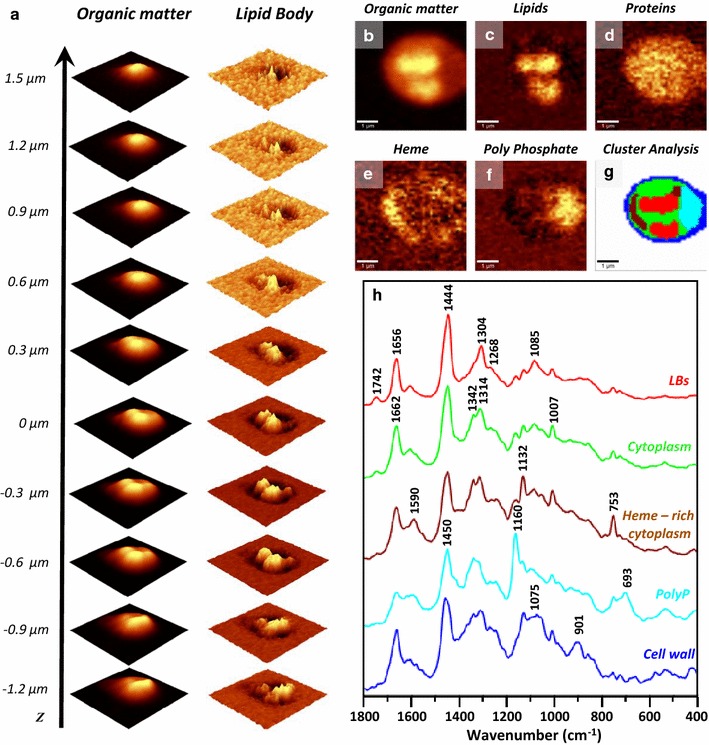
Comprehensive analysis of individual HBY31 cells via confocal Raman spectroscopy. **a** Confocal imaging enables depth profiling showing the distribution of selected components. The depth profiling was done with a step size of 300 nm. The distribution of *Organic matter* is demonstrated on the basis of integration of bands in the range 3050–2800 cm^−1^ and visualises the cell. The integration of the band at 1444 cm^−1^ enables us to visualise *LBs*. As can be seen, the LBs’ signals are present only within selected layers, corresponding to the inside of cell. **b**–**f** Distribution of selected components in a cell with polyphosphate vacuole, on the basis of integration of bands at: (**b**) 3050–2800 cm^−1^ (‘*Organic matter*’), (**c**) 1444 cm^−1^ (‘*Lipids*’), (**d**) 2925 cm^−1^ (‘*Proteins*’), (**e**) 753 cm^−1^ (‘*heme*’), (**e**) 1160 cm^−1^ (‘*Polyphosphate*’). **g**, **h** Cluster analysis results obtained for the cell presented in (**b**–**f**): (**g**) distribution of classes and (**h**) corresponding spectra of each class with marked selected bands. Size of the imaged area: (**a**) 5.32 × 5. 24 µm and (**b**) 5.22 × 5.17 µm. With the use of 532 nm excitation wavelength the theoretical thickness of each plane, according to Rayleigh criterion, is 361 nm

By imaging the cell in different planes, a three-dimensional chemical profile was mapped with the lipid-related bands, attributed to LBs, clearly visible within the cell (Fig. 4a). Via selection of the appropriate plane inside the cell covering a thickness of ~ 300 to 400 nm and spatial identification of LBs within this plane, spectra were obtained from LBs only. Simultaneously, all other cellular structures were studied by integration of marker bands related to their chemical components (Fig. 4b–f) and cluster analysis performed (Fig. 4g) from all spatially localised and averaged spectra obtained from subcellular structures (Fig. 4h). As can be seen, the LBs (Fig. 4b, g, h: red) contained bands attributed to SFAs (1444, 1304 cm^−1^), UFAs (1656, 1268 cm^−1^), TAGs (1742 cm^−1^) and phospholipids (1085 cm^−1^). The CRS spectrum of cell walls (Fig. 4g, h: dark blue) had a significantly different profile, characteristic of carbohydrates (e.g., 901, 1075 cm^−1^) with some protein contributions (1662 cm^−1^). Cytoplasm (Fig. 4d, g, h: green) consisted mainly of protein (1662, 1342, 1314 cm^−1^), including, e.g., phenylalanine (1007 cm^−1^) and heme. Within the cytoplasm, areas of high heme content (1590, 1132, 753 cm^−1^) can be identified (Fig. 4e, g, h: brown). Heme (iron protoporphyrin IX) is an essential molecule for yeast; it serves as a prosthetic group in enzymes and proteins especially those involved in transporting oxygen or in oxidation reactions in addition to its many roles in cell signaling, etc. Furthermore, cluster analysis revealed the presence of a large structure (Fig. 4f–h: light blue), characterised by marker bands at 1160 and 693 cm^−1^, attributed to inorganic polyphosphate (PolyP) [22]. PolyP has been reported to accumulate in a variety of organisms including yeast at up to 20% of dry cell weight [29] and while some biological functions of PolyP are known, its exact physiological role remains unclear. Here, PolyP accumulations were observed only in the most highly engineered cell line (HBY31).

### Lipid body characteristics and fatty acid composition of individual engineered yeast cells

**Fig. 5:**
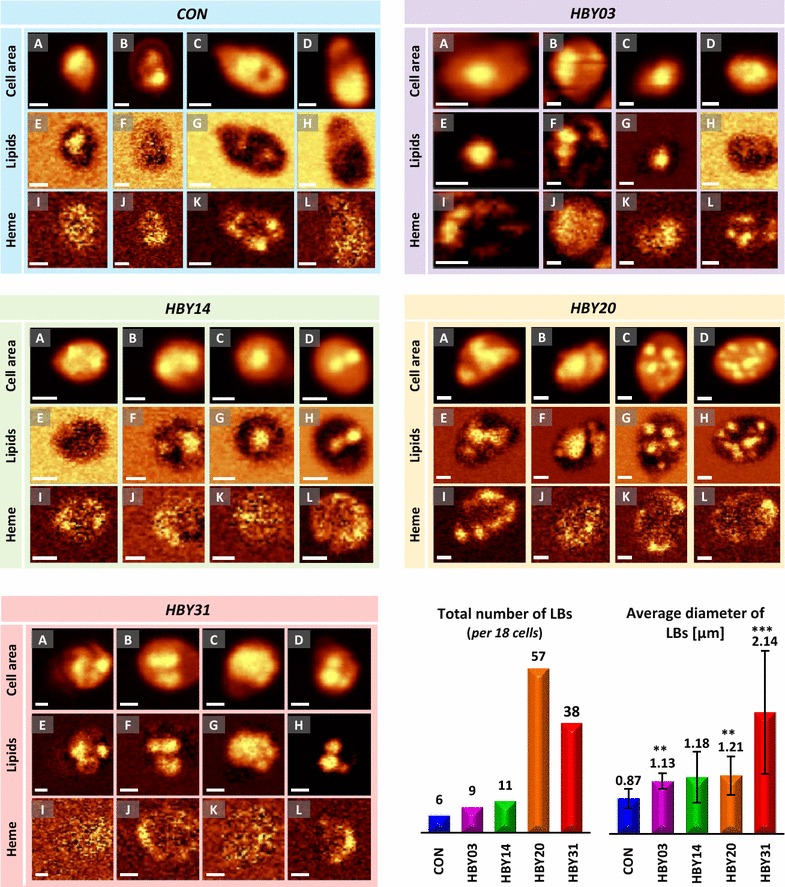
Distribution of selected components and LBs in yeast cells obtained via confocal Raman spectroscopy. In all panels: A–D the cell area is visualised by integration of the range 3050–2800 cm^−1^ (‘*Cell area’*); E–H LBs are shown by integral intensity of the band at 1444 cm^−1^ (‘*Lipids*’), I–J heme presence is shown by integral intensity of the band at 753 cm^−1^. Results of analysis of the presence of selected components are shown for single cells of all yeast cell lines: CAV (blue), HBY03 (purple), HBY14 (green), HBY20 (orange) and HBY31 (red). Each panel contains results obtained for 4 cells [in each panel: (A, E, I) cell 1, (B, F, J) cell 2, (C, G, K) cell 3, (D, H, L) cell 4]. The scale bar corresponding to 1 µm is shown in the bottom left of each image. The total number of observed LBs (measure in 18 cells per strain) and the summary of average diameter of LBs in each strain (± SD) are shown at bottom right. Detailed measurements for LBs are given in Additional file 1: Table S2. ***p < 0.01 vs control and **p < 0.05 vs control

To investigate the variability in presence of LBs, their size and composition within the same metabolic engineered line and between the cell lines, CRS imaging was applied to a total of 90 cells drawn equally from all strains. The distribution of selected components (organic matter, lipids and heme) from a subgroup of these cells is shown in Fig. 5 together with the total number of observed LBs in each cell line and their average diameters. In the case of the control, HBY03 and HBY14 strains show single LBs (per cell) with a diameter of ~ 1 µm, or lacking obvious LBs, with a minor increase in the number of LBs in the engineered lines compared to control. A significant increase in the number of LBs in cells of HBY20 and HBY31 was observed, with HBY20 having the highest among all strains and with LBs present in every cell.

Interestingly, LBs in HBY20 were only slightly increased in size (diameter 1.21 ± 0.40 µm) compared to control (diameter 0.87 ± 0.23 µm) (Fig. 5). For HBY31 strain, LBs were observed in all cells, however, in two types of arrangements. Some cells, similarly to HBY20, contained multiple LBs with a diameter of approximately 1 µm, but others contained a single large LB which filled the cell almost entirely (Fig. 5, HBY31 Panel G). This resulted in fewer but overall significantly larger LBs in HBY31 (2.14 ± 1.08 µm) compared to any other strain. HBY31 was the only strain assessed in this study that expressed caleosin (Table 1), a lipid droplet stabilizing protein originating from plants which may account for the larger LBs observed here.

**Fig. 6:**
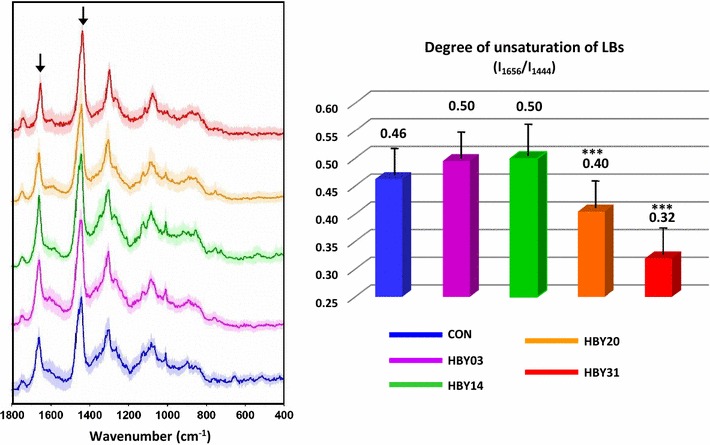
Assessment of the degree of unsaturation of LBs in engineered yeast strains. (Left) Average Raman spectra from all measured LBs (per cell line) together with their standard deviation, in the range 1800–400 cm^−1^. Black arrows mark the bands used for calculation of the degree of unsaturation. (Right) Degree of unsaturation of LBs calculated as the ratio of the band at 1656 cm^−1^ (C=C) to the band at 1444 cm^−1^ (CH_2_) (average value ± SD). ***p < 0.01 vs control

For a detailed investigation of LB composition, average spectra of each LB from each cell line (calculated from spectra of single LBs) were generated and variability measured (Fig. 6). Spectra of LBs contain several bands in the fingerprint region that are attributed to UFAs (1656, 1268 cm^−1^) and SFAs (1444, 1304 cm^−1^) with the ratio of intensities of I_1656_/I_1444_ being among the best indicators of fatty acid unsaturation [30]. An alternative approach for measuring fatty acid unsaturation by comparing intensities at 3012 and 2855 cm^−1^ is much less sensitive to unsaturation changes. No significant difference was found in the degree of unsaturation in LBs from HBY03 and HBY14 strains compared to control, but a significant decrease in unsaturation of LBs was observed for HBY31 (0.32 ± 0.05), and to a lesser extent HBY20 (Fig. 6). The observed decrease in fatty acid unsaturation was unlikely to be due to the larger size of LBs in HBY31 as the reduction was also observed in HBY20 for which no change in LB size was noted. This result indicated a shift to higher saturated fatty acid content in LBs in the more engineered strains measured via spectroscopy and the result was supported by GC analysis of fatty acid composition (Additional file 1: Table S1). It should be noted that CRS analysis was specifically measured in LB whereas GC analysis encompassed the whole cell fatty acid composition.

The shift to higher saturated fatty acid content in the most highly engineered strains was not only due to the specificity of the introduced acyltransferase, DGAT1, for saturated fatty acids as the *AtDGAT1* gene was expressed in all strains but maybe also due to limited quantities of unsaturated fatty acids available for conversion to triacylglycerol. ∆9-Unsaturated fatty acids are essential components of plasma membranes of yeast [31] and may be preferentially directed to these locations potentially resulting in more saturated fatty acids directed to triacylglycerol production through the enhanced expression of diacylglycerol acyltransferases.

### Comprehensive mapping of cellular constituents by AFM–IR

To obtain a comprehensive measure of biochemical changes in cells resulting from induced genetic modifications, further analysis of the composition of cytoplasm and, in particular, carbohydrate content was required. The ability to simultaneously study the composition of intracellular structures without the need to isolate them is an advantage of high spatial imaging via vibrational spectroscopy. However, as described earlier, the cytoplasm of engineered yeast contains significant and varying amounts of heme in the form of clusters and dispersed within the cytoplasm (Fig. 4e, g, h-Brown). The raised and variable Raman background due to heme content effectively conceals less intense signals such as carbohydrate-related bands excited at lower wavelengths and therefore, IR-based spectroscopy is more useful to investigate carbohydrate components at high spatial resolution. However, as the spatial resolution of conventional IR imaging does not exceed ~ 5 µm, a more sophisticated approach based on AFM–IR imaging was applied in our case. In AFM–IR the phenomenon of IR absorption is not measured directly, but by measuring the thermal expansion of the sample resulting from application. Therefore, the spatial resolution of imaging with the use of AFM–IR is no longer limited to ~ 5 µm, but can be significantly smaller (~ 100 nm), enabling the measurement of spectra representing explicitly the composition of subcellular structures. AFM–IR mapping with selected bands corresponding to proteins, lipids and carbohydrates was performed on 6 replicate cells for each strain and the images were combined and analysed via cluster analysis to confirm presence (or absence) of LBs and determine their precise location within the cell. Subsequently, single spectra (5–10) were collected from areas corresponding to LBs and cytoplasm. A high prevalence of LBs in the HBY31 and HBY20 strains were observed, in agreement with results shown previously by other techniques applied in this study.

**Fig. 7:**
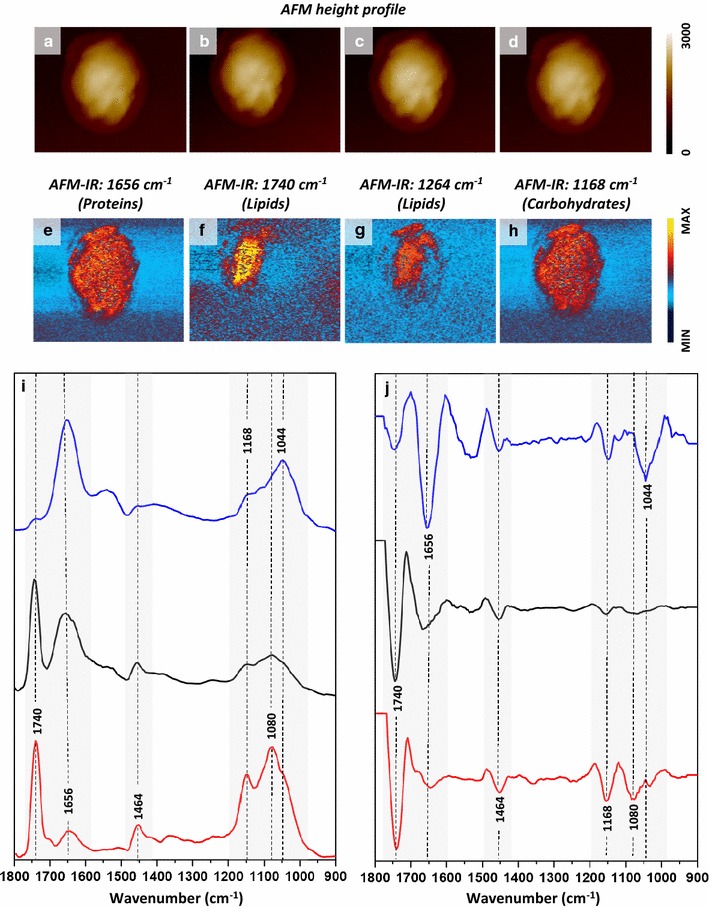
AFM–IR analysis of HBY31 cells. **a**–**d** AFM height results recorded simultaneously with **e**–**h** AFM–IR imaging of selected wavenumbers, demonstrating the distribution of chosen components: **e** proteins (1656 cm^−1^) (corresponding height: **a**), **f** lipids (1740 cm^−1^) (corresponding height: **b**), **g** lipids (1264 cm^−1^) (corresponding height: **c**) and **h** carbohydrates (cell wall, 1168 cm^−1^) (corresponding height: **d**). The size of imaged area was 5.41 × 4.47 µm. The scale bar for AFM height is given in nm. **i** A comparison of AFM–IR spectra and **j** their 2nd derivatives in the range 1800–900 cm^−1^ recorded from: LB (in red) of HBY31 cell, cytoplasm of HBY31 cell (in black) and cytoplasm of control cell (in blue). The spectral regions and bands showing most prominent differences were marked in grey background and black dash lines

Focussing on HBY31 strain, Fig. 7a–d shows the AFM height profile recorded simultaneously with each AFM–IR map and the relatively even thickness of the cell (approximately 3 µm). The presence of LBs was revealed through imaging of the absorbance at 1740 cm^−1^ (stretching of C=O from lipids) (Fig. 7f) and 1264 cm^−1^ (deformation of CH_2_ of lipids) (Fig. 7g). The protein (Fig. 7e) and carbohydrate (Fig. 7h) distribution in the cells were homogenous, as these signals originate from both cytoplasm and cell wall, with the latter being of even thickness across the cell. A comparison between AFM–IR spectra of LB (Fig. 7i, red) and cytoplasm (Fig. 7i, black) of the same HBY31 cell showed large differences, the LB spectrum was dominated by lipid-related signals (1740, 1464, 1080 cm^−1^) and an intense phospholipid band (1080 cm^−1^, stretching of PO_2_) originating from the LB membrane. The spectra of the HBY31 cytoplasm (Fig. 7i, black) has higher protein (1656 cm^−1^) to lipid (1740 cm^−1^) ratio compared with the LB spectra (Fig. 7i, red). A comparison of 2nd derivatives of AFM–IR spectra of cytoplasm from both HBY31 (Fig. 7j, black) and control (Fig. 7j, blue) showed interesting differences: although the HBY31 cytoplasm shows lower intensity due to lipids than LB from the same cell, it reveals a higher lipid content in the cytoplasm, compared to the control cell (Fig. 7j, blue). In terms of metabolic engineering, the measurement of significant quantities of lipid in the cytoplasm suggests the lipid accumulation and sequestration strategies for HBY31 cells are out of balance with production, and lipid storage capacity of LBs in this strain has been overwhelmed. In addition, a band of significant intensity at 1044 cm^−1^ in control cells (Fig. 7j, blue) attributed to carbohydrate, was absent in the cytoplasm of HBY31 (Fig. 7j, black), suggesting an acute reduction of the carbohydrates in favour of enrichment of lipids in the cytoplasm of HBY31.

### AFM–IR reveals cell-to-cell variability in concentrations of key metabolites

**Fig. 8:**
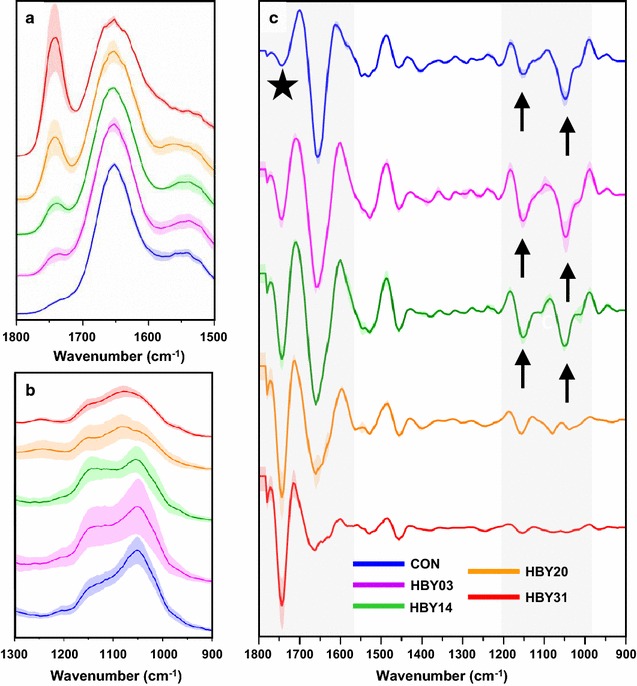
Averaged AFM–IR spectra of cytoplasm of engineered yeast cells in the range (**a**) 1800–1500 cm^−1^ and (**b**) 1300–900 cm^−1^ together with **c** their 2nd derivative in the range 1800–900 cm^−1^. Each presented average spectrum was obtained by averaging 30 single spectra collected for different cells (of the same strain) and is shown with SD. Each single spectrum in this study was collected from cytoplasm, after localization of the LB (or confirmation of its absence) through AFM–IR imaging. Grey area, black star and black arrows mark the most prominent differences

The AFM–IR spectra obtained from individual cells in all strains were analysed with respect to the relationship between carbohydrate and lipid content, and variance between samples. The average spectra for cytoplasm for each strain are shown in Fig. 8 together with the variance within the strain. The lipid content in the cytoplasm (e.g., 1740 cm^−1^) undoubtedly increases, from low levels in control cells, through higher amounts for HBY03 and HBY14, to very high concentrations for HBY20 and HBY31 cell lines (Fig. 8a, c marked with a star), and the intra-strain variability in cytoplasm lipid increases. Concurrent with the increase of the intensity of lipid-related bands with more engineered strains, was a decrease in intensity of the bands in the range 1100–900 cm^−1^ (Fig. 8b). A detailed comparison of 2nd derivative spectra revealed the contribution of different components in this region: for control, HBY03 and HBY14, the carbohydrate-related band at 1044 cm^−1^ was clearly present (Fig. 8c, black arrows) and of significant intensity. For cytoplasm of HBY20, the carbohydrate band was just visible but substantially lower and the emergence of the phospholipid band at 1080 cm^−1^ was evident (Fig. 8b). As discussed previously, the cytoplasm of HBY31 cells had almost no intensity at 1044 cm^−1^ whereas the band at 1080 cm^−1^ became more prominent (Fig. 8b). Altogether, the AFM–IR results demonstrate the increase in lipid content and a simultaneous decrease in the carbohydrate content in the cytoplasm, from high carbohydrate and low lipid in control cells to the reversed ratio in the HBY31 strain.

Vibrational spectroscopy analysis has previously been shown to be highly informative for measurement and location of target metabolites such as lipids in cultured cells of wild-type microalgae and yeast [17, 19, 32–40] and in selected studies that include heterologous expression of lipid stabilizing proteins [41]. Our study is the first to our knowledge where the effects of the sequential introduction of a complex metabolic engineering pathway into an organism have been measured using vibrational spectroscopy techniques and have revealed valuable insights into areas for further improvement.

## Conclusions

Here, we have used vibrational spectroscopy techniques to measure the impact of metabolic engineering approaches for high lipid production in yeast at both the single cell level and averaged and detailed information about the chemical composition of subcellular structures. The ratio of lipid-related bands (e.g., 1740 cm^−1^) to amide I was demonstrated to be useful as a quick marker of the total lipid content. An established PLS regression model allowed the successful prediction of total fatty acid content from the ATR-FTIR spectrum, demonstrating the ability of this technique to provide the same information as GC, but within few minutes and without the need for complex sample preparation. Fluorescence imaging enabled us to visualise LBs, confirming their highest prevalence and size in the HBY31 strain. Furthermore, two high spatial imaging techniques based on vibrational spectroscopies were applied (CRS, AFM–IR) and demonstrated substantial changes in intracellular composition with more complex metabolic engineering. CRS imaging provided an insight into LBs composition, revealing a significant decrease in the degree of unsaturation of lipids in the most highly engineered strain—HBY31. In addition, CRS imaging demonstrated significantly increased LB number in HBY20 and HBY31 strains together with a dramatic increase in LB size for HBY31 that expressed the LB stabilizing protein, caleosin. Finally, the AFM–IR imaging demonstrated large changes in the composition of cytoplasm between strains. A decrease in carbohydrate content with concurrent increase in lipid content of cytoplasm was observed, progressing from control through to HBY31 strains. The high concentration of lipid in the cytoplasm of HBY31, in particular, suggests lipid production rate in these engineered yeast is exceeding the rate of lipid sequestration in LBs which could lead to lipotoxicity. Additionally, the shift towards higher saturated fatty acids levels in stored lipids suggests a reduction in the availability of unsaturated fatty acids for TAG formation in the cells. Altogether, our results have demonstrated an increase in the total lipid content resulting from genetic modifications, with the multigene modification approach (*Ald6, SEACS*^*L641P*^*, ACC1*^*S659A, S1157A*^*, AtDGAT1, AtClo1* and *Tgl3△*) in the HBY31 cell line being most effective. The vibrational spectroscopy approach allowed the simultaneous measurement of intra-strain variability in metabolite production and impact on cellular structures from metabolic engineering.

## Additional file

**Additional file 1.** Supplementary Information.

## Abbreviations

ACC: acetyl-CoA carboxylase
ACS: acetyl-CoA synthetase
AFM–IR: atomic force microscopy–infrared spectroscopy
ALD: aldehyde dehydrogenase
AtClo1: caleosin from *Arabidopsis thaliana*
ATR-FTIR: attenuated total reflection-Fourier transform infrared spectroscopy
CRS: confocal Raman spectroscopy
DCW: dry cell weight
DGAT1: diacylglycerol acyltransferase
FA: fatty acid
FAME: fatty acid methyl ester
GC: gas chromatography
GC-FID: gas chromatography with flame ionization detector
IR: infrared (spectroscopy)
LB: lipid body
PBS: phosphate buffer solution
PLS: partial least square
PolyP: inorganic polyphosphate
RS: Raman spectroscopy
SFA: saturated fatty acid
SNV: standard normal variate
TAG: triacylglycerol
Tgl3: triacylglycerol lipase 3
UFA: unsaturated fatty acid
