# Supplementary material for: Single cell assessment of yeast metabolic engineering for enhanced lipid production using Raman and AFM-IR imaging
Source: Biotechnol Biofuels. 2018 Apr 10;11:106. doi: 10.1186/s13068-018-1108-x (PMC5891968; doi:10.1186/s13068-018-1108-x)
Supplement: Supplementary file 1 — Additional file 1. Supplementary Information. [file 13068_2018_1108_MOESM1_ESM.docx]

**Additional materials**

For submission to*: Biotechnology for Biofuels*Section: *Research*

***Single cell assessment of yeast metabolic engineering for enhanced lipid production using Raman and AFM-IR imaging***

**Kamila Kochan^1†^, Huadong Peng^2†^, Bayden R. Wood^1^, Victoria S. Haritos^2,*^**

^1^Centre for Biospectroscopy, School of Chemistry, Monash University, Clayton Campus, 3800, Victoria, Australia

^2^ Department of Chemical Engineering, Monash University, Clayton Campus, 3800, Victoria, Australia

**^†^**Equal contributors

Author for correspondence:

Assoc Prof Victoria Haritos,
Department of Chemical Engineering,
Monash University, Clayton 3800, Victoria Australia

Phone: +61 3 9905 6873
email: victoria.haritos@monash.edu

**Table S1**. GC analysis of total fatty acid content in yeast strains.

| **Sample** | **Total fatty acid content**  **[% of dry cell weight]** | | **Fatty acid composition**  **[% of total fatty acid content]** | | | | | | |
| --- | --- | --- | --- | --- | --- | --- | --- | --- | --- |
|  | **Mean** | **SD** | **C10:0** | **C12:0** | **C14:0** | **C16:1** | **C16:0** | **C18:1** | **C18:0** |
| **CON** | 3.08 | 0.22 | 0.00 | 0.61 | 0.00 | 54.67 | 19.06 | 17.14 | 8.52 |
| **HBY03** | 4.10 | 0.19 | 0.00 | 0.88 | 1.32 | 47.47 | 22.04 | 16.59 | 11.70 |
| **HBY14** | 5.90 | 0.25 | 1.28 | 1.24 | 1.97 | 45.25 | 23.55 | 16.26 | 10.44 |
| **HBY20** | 7.12 | 0.75 | 1.80 | 1.54 | 2.61 | 38.30 | 28.96 | 14.09 | 12.71 |
| **HBY31** | 7.97 | 0.87 | 1.92 | 1.63 | 2.82 | 37.68 | 29.59 | 13.53 | 12.84 |

**Table S2**. The average diameter (μm) of LBs in yeast strains together with standard deviation (SD) measured by confocal Raman spectroscopy. (The values are represented as a bar chart in Fig. 5.)

| **CON** | | **HBY 03** | | **HBY 14** | | **HBY 20** | | **HBY 31** | |
| --- | --- | --- | --- | --- | --- | --- | --- | --- | --- |
| ***Mean*** | ***SD*** | ***Mean*** | ***SD*** | ***Mean*** | ***SD*** | ***Mean*** | ***SD*** | ***Mean*** | ***SD*** |
| 0.87 | 0.23 | 1.13 | 0.16 | 1.18 | 0.53 | 1.21 | 0.40 | 2.14 | 1.08 |

**
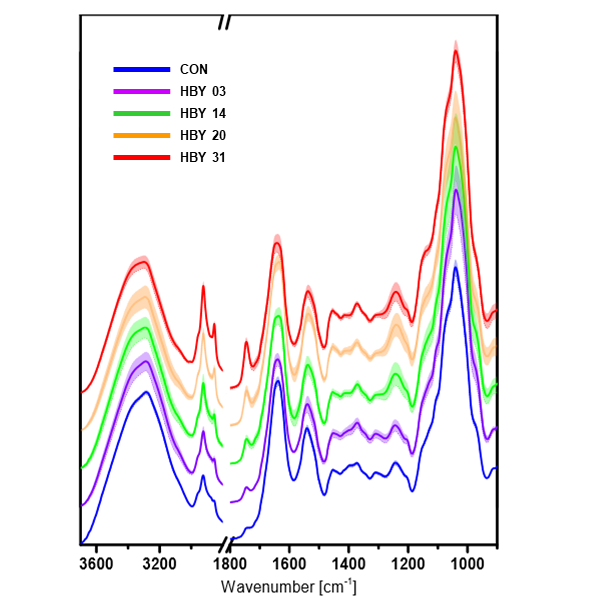
**

**Figure S1.** ATR-FTIR average spectra (and SD) of all measured cell lines in the range 3700 – 900 cm^-1^. Each spectrum is an average of 9 single spectra (3 technical replicates of 3 biological replicates.).


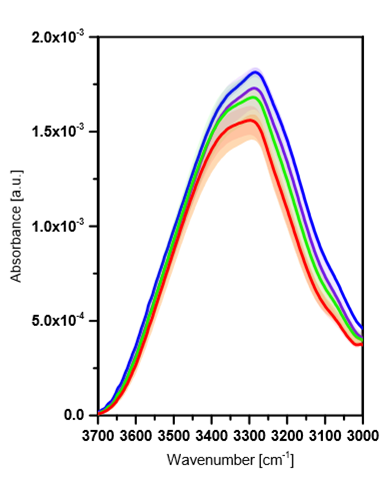


**Figure S2.** ATR-FTIR average spectra (and SD) of all measured cell lines in the range 3700 – 3000 cm^-1^. Each spectrum is an average of 9 single spectra (3 technical replicates of 3 biological replicates.). Colour coding of spectra is presented in Figure S1.
